# Supplementary material for: The effect of COVID-19 vaccine tele-educational program on vaccine hesitancy and receiving the vaccine among women planning for pregnancy, pregnant or breast-feeding mothers
Source: PLoS One. 2023 Mar 9;18(3):e0282627. doi: 10.1371/journal.pone.0282627 (PMC9997991; doi:10.1371/journal.pone.0282627)
Supplement: Attachment — Submitted filename: Response to Reviewers.docx [file pone.0282627.s002.docx]

Title: The effect of COVID-19 vaccine tele-educational program on vaccine hesitancy and receiving the vaccine among women planning for pregnancy, pregnant or breast-feeding mothers

Dear all, thank you for your valuable comments, I am delighted that your comments contributed to the manuscript, please see the table below.

| **Comments** | **Responses** |
| --- | --- |
| please adhere to the submission guidelines regarding spacing in the article format | Fixed and referencing was changed to adhere with the guidelines |
| Please Include page numbers and line numbers in the manuscript file. | Page and line numbers are included |
| please use the correct referencing style as outlined in the submission guidelines | Corrected |
| please correct the Manuscript Organization style of the manuscript as per the submission guidelines | Fixed accordingly |
| correct the following spelling mistakes:  page 5, line 4 vaccination instead of vaccines  page5, line 10 change declining to decline  page6, line 7 correct spelling of COVID-19  page6, line 32 change Statisitics to Statistics | Corrected |
| regarding introduction: please consider Include a brief review of the key literature and making it more precise and concise, with less wording. | Rephrased and corrected accordingly |
| avoid overstating the conclusions. Authors may discuss possible implications for their results as long as these are clearly identified as hypotheses instead of conclusions | Fixed |
| References are listed at the end of the manuscript and numbered in the order that they appear in the text. In the text, cite the reference number in square brackets. | Done |
| make sure to read the submission guidelines in details and correct the outline of the manuscript | Done |
| We recommend that authors seek independent editorial help before submitting a revision. These services can be found on the web using search terms like “scientific editing service” or “manuscript editing service. | The manuscript has now proof read |
| please consider adding the following articles of a similar topic and discussed the topic you are discussing and published form Jordan:  A. Aloweidi A, Bsisu I, Suleiman A, et al.: Hesitancy towards COVID-19 vaccines: an analytical cross-sectional study. Int J Environ Res Public Health. 2021, 18:5111. 10.3390/ijerph18105111  B. Abdullah M, Shahait A D, Qaisieh R, et al. (June 26, 2022) Perspectives on COVID-19 Vaccines and Its Hesitancy Among Jordanian Population. Cureus 14(6): e26337. doi:10.7759/cureus.26337 | Added to the introduction |
| 1. Please ensure that your manuscript meets PLOS ONE's style requirements, including those for file naming | Done |
| 2. We note that the grant information you provided in the ‘Funding Information’ and ‘Financial Disclosure’ sections do not match.  When you resubmit, please ensure that you provide the correct grant numbers for the awards you received for your study in the ‘Funding Information’ section. | Fixed accordingly |
| 3. Thank you for stating the following in the Funding statement Section of your manuscript:  "This research project was funded by the Applied Science Private University, Amman, Jordan."  We note that you have provided funding information that is not currently declared in your Funding Statement. However, funding information should not appear in the Acknowledgments section or other areas of your manuscript. We will only publish funding information present in the Funding Statement section of the online submission form.  Please remove any funding-related text from the manuscript and let us know how you would like to update your Funding Statement. Currently, your Funding Statement reads as follows:  "The funder had no role in study design, data collection and analysis, decision to publish, or preparation of the manuscript."  Please include your amended statements within your cover letter; we will change the online submission form on your behalf. | Corrected accordingly |
