# Supplementary material for: The effect of COVID-19 vaccine tele-educational program on vaccine hesitancy and receiving the vaccine among women planning for pregnancy, pregnant or breast-feeding mothers
Source: PLoS One. 2023 Mar 9;18(3):e0282627. doi: 10.1371/journal.pone.0282627 (PMC9997991; doi:10.1371/journal.pone.0282627)
Supplement: Attachment — Submitted filename: Response to reviewers.docx [file pone.0282627.s003.docx]

**Title:** The Effect of COVID-19 Vaccine Tele-educational Program on Vaccine Hesitancy and Receiving the Vaccine Among Women Planning for Pregnancy, Pregnant or Breast-feeding Mothers

Dear all, thank you for your valuable comments, please see the table below, it is the response from the authors.

| **Reviewers’ Comments** | **Authors’ Responses** |
| --- | --- |
| Please review your reference list to ensure that it is complete and correct. If you have cited papers that have been retracted, please include the rationale for doing so in the manuscript text, or remove these references and replace them with relevant current references. Any changes to the reference list should be mentioned in the rebuttal letter that accompanies your revised manuscript. If you need to cite a retracted article, indicate the article’s retracted status in the References list and also include a citation and full reference for the retraction notice. | Several references were added and references were updated accordingly. |
| **Reviewer 1** |  |
| Although the language needed to be enhanced to a standard level. Data availability section should be added. | Language is checked. And data availability statement was added. |
| This study has been performed in 2021, I suggest the authors should go and see the state of the art in the topic and enrich the introduction and discussion. | Updated |
| The sample is not quite representative as doesn't include women from south Jourdan. | Participants were sampled from the three major governorates in Jordan |
| **Reviewer 2** |  |
| Introduction: 1. The first paragraph of the Introduction is too long. Thus, I encourage the authors to divide the Introduction into 2 or 3 paragraphs rather than one paragraph. For example: The second paragraph could be started with “Global studies found that pregnant women ………” The third paragraph from “Based on risk-benefit calculations……….” | Done |
| 2. Also, I prefer to include in the first paragraph of the Introduction section more information about seroprevalence and vaccine coverage worldwide and in developing countries. Also, you could mention the benefits of vaccination over traditional precautionary measures, such as curfews, isolation, sealing the borders …..etc. These benefits could be included in the Introduction and Discussion sections of the manuscript. You could use the following references … | Considered |
| Methods: 3. In the Ethical considerations subsection, could you clarify the organizations from which participants were recruited? | Added to the ethics section |
| 4. Did the participants receive any compensation or rewards for participating in the study? Please, include this point in the manuscript. | A sentence was added for clarification |
| 5. Are there any missing data during the collection process? If yes, how did you deal with it? | There was missing data of the main variable of the study for 6 cases. Those were excluded from the analysis. A sentence was added for clarification. |
| 6. Do the authors have data about the number of women who refused to participate and their reasons? | 5 women refused to participate without giving a reason. A sentence was added for clarification. |
| Results: 7. In the first paragraph, you mentioned again, “Before the program, none of them received the COVID-19 vaccine.” This point is redundant for the inclusion criteria, and I prefer to remove it here. | Removed |
| 8. Could you calculate and add the p-values to table 1 to compare the two groups? Also, please raise up the age variable in table 1 to the third raw (Directly after the group variable) | Table 1 is only a description table. A p-value was added to compare groups in table 2, 3 and 4. |
| 9. This paragraph is redundant for what was previously mentioned in the Methods: “Three analysis steps were done to determine the effect of a tele-educational program on vaccine hesitancy among women planning for pregnancy and pregnant and breast-feeding mothers. Firstly, an independent t-test was used to examine any difference in the mean score of the hesitancy to get the vaccine before conducting the program between the intervention group and the control group” >>> The Results section should include the study findings with no details on the study strategy and analysis steps. These points should be just mentioned in the Methods section of the manuscript. | Removed |
| 10. Similarly, the following paragraph should be omitted from the Results section: “Secondly, an independent t-test was used to examine any difference in the mean score of the hesitancy to get the vaccine between the intervention group and the control group after conducting the program.” | Removed |
| 11. In table 2, the p-value of 000 should be presented as (<0.001). As well as, the p-values in table 3 and table 4 should be corrected. | Fixed |
| 12. Also, the following paragraph is redundant and should be omitted from the Results section: “Thirdly, a paired samples t-test was conducted to examine any difference in the mean score of the hesitancy to get the vaccine for the intervention group and control group before and after the program.” | Removed |
| 13. This paragraph should be transferred from the Results section to the Methods section: “Concerning COVID-19 vaccination, related samples McNemar test was conducted to examine any vaccine rate difference before and after the program.” | Moved |
| 14. The last paragraph in the Results section should be transferred to the Discussion and Conclusion sections of the manuscript: “To conclude, there was no significant difference in the hesitancy of COVID-19 vaccination …………………… of COVID-19 vaccination and increasing the tendency to get the vaccine in the interventional group only.” | It was removed to avoid repetition |
| Discussion: 15. The discussion should be extended with more details to include more information about vaccination hesitancy and determinants in developing countries, particularly among women planning to be pregnant, and how this study will solve this significant issue. Also, as previously mentioned, I prefer to include the benefits of vaccination over traditional precautionary measures, such as curfews, isolation, sealing the borders …..etc. In addition, this study’s findings should be compared with previous studies in the literature. I would refer you to the following helpful references … | Considered |
| 17. Study limitations: A. The study sample size is relatively small. However, the sample was collected from different areas throughout the country. B. The selection bias cannot be excluded as the study participants were collected from prenatal/maternity clinics. Thus, the results are unlikely to be generalizable beyond those who responded. C. Also, the point mentioned in the manuscript: “Although the study aimed to include only pregnant women, planning for pregnancy, and breast-feeding mothers, their answers may vary from the general population and people in other situations.” | Fixed accordingly |
